# Alleviating the adverse effects of salinity on Roselle plants by green synthesized nanoparticles

**DOI:** 10.1038/s41598-022-22903-9

**Published:** 2022-10-28

**Authors:** Mohammad Sadat-Hosseini, Atena Naeimi, Naser Boroomand, Mostafa Aalifar, Mostafa Farajpour

**Affiliations:** 1 Department of Horticultural Science, Faculty of Agriculture, University of Jiroft, Jiroft, Iran; 2 Department of Chemistry, Faculty of Science, University of Jiroft, Jiroft, Iran; 3 Department of Soil Science, Faculty of Agriculture, Shahid Bahonar University of Kerman, Kerman, Iran; 4 Young Researchers and Elite Club, Hamedan Branch, Islamic Azad University, Hamedan, Iran; 5 Crop and Horticultural Science Research Department, Mazandaran Agricultural and Natural Resources Research and Education Center, Agricultural Research, Education, and Extension Organization (AREEO), Sari, Iran

**Keywords:** Plant stress responses, Salt, Biotechnology

## Abstract

In the present study, an eco-friendly process was made for the rapid synthesis of silver nanoparticles using aqueous leaf extract of *Hibiscus sabdariffa*. The process was characterized by Fourier Transform Infrared (FT-IR), scanning electron microscopy (SEM), transmission electron microscopy (TEM), UV–visible and X-ray diffraction (XRD). These green silver nanoparticles (NPs) were used for mitigating the adverse effects of salinity on seed germination and growth parameters in plants. Accordingly, two experiments were conducted. In the first experiment, seven concentrations of green silver NPs and nine levels of NaCl:CaCl were apptoed on seeds for germination, and their effects were evaluated. In the second experiment, three concentrations of green silver NPs and NaCl were hypothesized to affect plant growth parameters. Seed germination, plant height, leaf, and root fresh and dry weights, as well as relative water content (RWC), decreased significantly under salt stress. However, green silver NPs intervened by alleviating the adverse effects of stress. Accordingly, green silver NPs were beneficial due to (1) activation of the antioxidant system by enhancing antioxidant enzymes such as catalase (CAT), ascorbate peroxidase (APX), peroxidase (POD), and superoxide dismutase (SOD); (2) increase in the amounts of proline, soluble sugars and carbohydrates for osmoprotection; (3) improvements in flavonoid and anthocyanin contents. Real-time PCR showed that flavonoid and anthocyanin contents increased because of higher expressions in chalcone synthase (*CHS*), flavanone 3‐hydroxylase (*F3H*), and anthocyanidin synthase (*ANS*) genes. In conclusion, green silver NPs offered an eco-friendly application for further research on agricultural development.

## Introduction

Roselle (*Hibiscus sabdariffa*) is commonly used as herbal medicine by indigenous people in Africa, India, Iran, Egypt, and Mexico^1^. Local people in these countries use the calyces or leaves to treat cholerectic, cardiac, and nerve diseases. The plant causes diuretic, febrifugal and hypotensive effects and reduces the viscosity of blood^2^. Although it originates in Asia, the species is widely cultivated in many regions, including Africa and Central America^3^. More than 20% of irrigated lands around the world can be seen as affected by excessive salinity, which can significantly reduce crop production. Excessive salinity generally leads to reductions in seed germination, photosynthesis, nutrient uptake, transpiration, and plant growth, thereby resulting in lower yield productions. Several plants are susceptible to high salinity during seed germination and seedling development^4^. While it is not clearly known why plants are susceptible to salinity during seed germination, germination can be usually affected by toxic levels of salinity. In addition, seeds may absorb smaller amounts of water when exposed to high levels of salinity^5^. Recently, nanoparticle materials have become a prevalent tool in agricultural endeavors, especially in seed germination^6^. Khodakovskaya et al.^7^ reported that carbon nanotubes were able to penetrate the seed coatomatoesomato and support water uptake inside seeds, thereby causing enhancements in seed germination and growth rates. The carbon nanotubes also improved the germinations of pepper (*Capsicum annum*) and salvia (*Salvia microsiphon*)^8^. Nano-sized silicon (N-Si) and nano-Ti are known to be capable of increasing nitrate reductase NR activity of soybean*.* Haghighi and Pessarakli^9^ reported that Si and N-Si were able to enhance mesophyll conductance, photosynthesis rate, and water use efficiency (WUE) of cherry tomatoes in saline soils. The applications of iron NPs improved growth-related parameters of strawberry and grape under salt stress^10^. These metal NPs were found to be capable of penetrating plant cells, which raises concerns that they can be hazardous for human health and the environment^11^. Currently, the green material/nanoparticle synthesis based on biocomponent-derived materials/nanoparticles is likely to be applied extensively both in the field of environmental remediation and in other important areas such as agricultural, pharmaceutical, food, and cosmetic industries^12,13^. Plant natural products encompass different secondary metabolites (SMs) such as flavonoids and phenolic compounds. The SMs enable the plant to decrease the metal ions to NPs in eco-friendly one-step synthetic processes^14^. Flavonoids and anthocyanin compounds are two of the most important secondary metabolic compounds in higher plants. They have multiple roles in plants such as growth, development, and reproduction, in addition to acting as antioxidants that scavenge reactive oxygen species (ROS). The accumulation of flavonoids and anthocyanin are associated with their response to abiotic and biotic types of stress. Few studies have dealt with the question of how the synthesis of flavonoids and anthocyanin may help plants develop tolerance against salt stress. Furthermore, chalcone synthase (CHS), flavanone 3‐hydroxylase (F3H), and anthocyanidin synthase (ANS) are three key enzymes in the biosynthetic pathway of flavonoids^15^. Salinity adversely affected plants in different ways such as seed germination and seedling growth. So far, there has been insufficient information about the application of NPs on Roselle.

Different regions in Iran are subject to soil salinity, which can put intense levels of stress on plants. Also, due to the medicinal properties of Roselle, the cultivation of this plant is becoming increasingly prevalent, so that vast areas are being allocated to its cultivation. Solving the salinity problem can effectively involve using green silver NPs. Therefore, the present study aimed to evaluate how the green synthesis of silver NPs can enhance seed germination and various growth stages in Roselle plants under salinity stress. In addition, this evaluation was accompanied by efforts to find the expression patterns of genes involved in the biosynthesis of flavonoids and anthocyanins.

## Materials and methods

### Green synthesis of silver nanoparticles

About 5 g of dried powder of *Hibiscus sabdariffa* were added to 200 mL distillate water. The extracted solution was filtered and stored at 4 °C^16^. The synthesis of silver NPs was carried out using the extract of *Hibiscus sabdariffa* and 20 mL silver nitrate (1 mM), which were added to a mixture of 12.5 mL water and 12.5 mL of the extracted solution. The reaction remained uninterrupted until a black color became noticeain the solution^17^.

**Figure 1:**
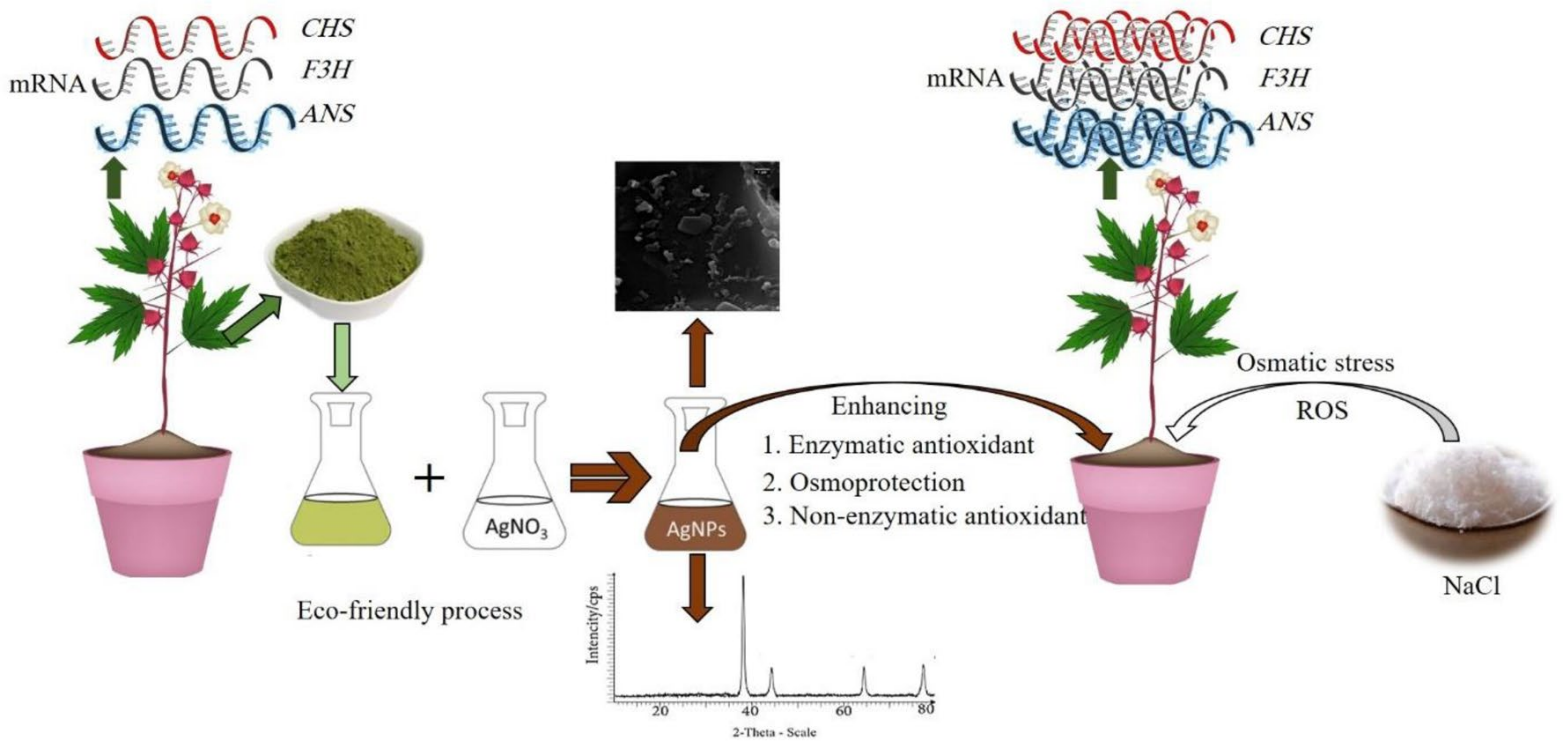
The schematic view of the step-by-step procedure of the present study.

In the present study, all methods were carried out in accordance with relevant guidelines and regulations. Step-by-step procedures of the experiment were followed accurately (Fig. 1).

### Seed germination

The seeds were sterilized by a commercial sodium hypochlorite solution (50% v/v) for 8 min, and were then rinsed with distilled water for 3 min, which was followed by a procedure of drying before transfer to petri dishes. The seeds were transferred to petri dishes which contained 6 mL of distillate water. The experiment was laid out as a factorial, according to the completely randomized design (CRD) with three replications. Two factors were used, including nine concentrations of NaCl:CaCl (1:1; 0, 15, 30, 45, 60, 90, 120, 150, and 180 mM) and seven levels of silver NPs (0, 15, 30, 45, 60, 75, and 90 mg/L).

The cultures were placed at 20 ± 1 °C. The distillate water was renewed every 2 days. Germination tests were conducted in three replicates and 30 seeds were placed in each petri dish.

### Growth parameters

In the second experiment, seedling growth parameters were evaluated under salinity and in the presence of silver NPs. When the seeds germinated, seedlings with similar sizes (having 4 leaves) were selected. The seedlings were transplanted into pots containing cocopeat/perlite (2/1 v/v). A solution of nutrients (mg L), containing HS-AgNPs, was added according to a method described in the literature^18^. A fresh solution of nutrients (150 mL) was poured into each pot every other day. To prevent plant shock, the salt was applied four times and the applications were distributed evenly over a total period of 10 days. This second experiment was also laid out as a factorial, based on a CRD design with three replications. Three concentrations of NaCl (0, 60, and 120 mM) and three levels of silver NPs (0, 45, and 90 mg/L) were evaluated on the growth parameters.

### Relative water content (RWC)

In order to measure RWC and other features, six individual plants were selected for each treatment. After harvesting the leaves, the fresh weights (FW) of eight leaf discs were recorded. The leaf discs were allowed to float on a water distillate for 12 h, and the turgid tissue was then quickly blotted dry, before determining the turgid weights (TW). To measure the dry weight (DW), leaf discs were dried at 80 °C for 72 h. The RWC was measured according to the following formula^19^:

$$ {\text{RWC}}\;(\% ) = ({\text{FW}} - {\text{DW}}) \times 100/({\text{TW}} + {\text{DW}}). $$

### Antioxidant enzymes activity, proline, and soluble sugars

Four antioxidant enzymes were recorded, including catalase (CAT), ascorbate peroxidase (APX), peroxidase (POD), and superoxide dismutase (SOD). Accordingly, 0.5 of the fresh leaf was ground in 5 mL of 50 mM phosphate buffer (pH 7.8) containing 1 mM EDTA and 1% soluble PVP. The solution was centrifuged at 13,000 rpm for 15 min at 4 °C. The CAT activity was monitored by the decline in absorbance at 240 nm due to the decrease in the extinction of H_2_O_2_ according to a procedure used by Patra et al.^20^. The APX activity was recorded based on the decrease in absorbance at 290 nm as ascorbate was oxidized^21^. The POD activity was determined by the increase in absorbance at 470 nm due to guaiacol oxidation. The SOD activity was measured by recording its capability of inhibiting the photochemical reduction of nitro blue tetrazolium. Proline content was quantified by using a relevant procedure available in the scientific literature^22^. The absorbance was measured at 520 nm using a UV–visible spectrophotometer. Soluble sugars were determined according to a relevant procedure^23^, whereby glucose was used as standard. The absorbance was determined at 485 nm by a spectrophotometer (PG instruments LTD).

### Total flavonoid and anthocyanin contents

A colorimetric that included aluminum chloride was used for determining the flavonoid content. In this study, quercetin was used as a standard. The total amount of anthocyanin was determined according to a method described by Lee et al.^24^. A buffer was prepared for the measurement of anthocyanin according to a relevant procedure^25^. Finally, the results were expressed as equivalents of cyanidin-3-glucoside per 100 g of leaf samples.

### Real-time PCR

The RNA was extracted from the leaves of Roselle (100 mg) using an RNeasy Mini Kit (Qiagen, Hilden). The DNase I (Qiagen) was used for eliminating genomic DNA contaminants. The quantity and quality of the RNA were estimated by spectrophotometry at 260 and 280 nm, respectively. First-strand complementary DNA (cDNA) was produced from 1 µg of total purified RNA. This included the M-MLV reverse transcriptase (Promega, Mannheim, Germany) and the oligo (dT) 20 primer.

The primers of *F3H*, *CHS*, and *ANS* genes were designed by the Vector NTI10 software (Invitrogen). The specificity of each pair of primers was also validated by randomly sequencing the PCR products. The primers with an amplification efficiency of more than 95% were selected for use in the present study. In order to avoid errors, the RT-PCR was typically normalized against the 18SrRNA housekeeping gene (Supplementary Table S1). The relative level of gene expression was determined by the 2^−ΔΔCt^ method^26^.

### Statistical analyses

In the present study, two different two-way ANOVA were used. In the first experiment, the effects of nine concentrations of salinity and six concentrations of NPs were evaluated on seed germination. In the second experiment, however, either the effect of salinity or NPs was assessed on seedling growth. In addition, the mean values were compared using the LSD test (P < 0.05). A heat map correlation analysis was visualized as a colored heat map using MetaboAnalyst.

## Results

### Characterization of silver nanoparticles

**Figure 2:**
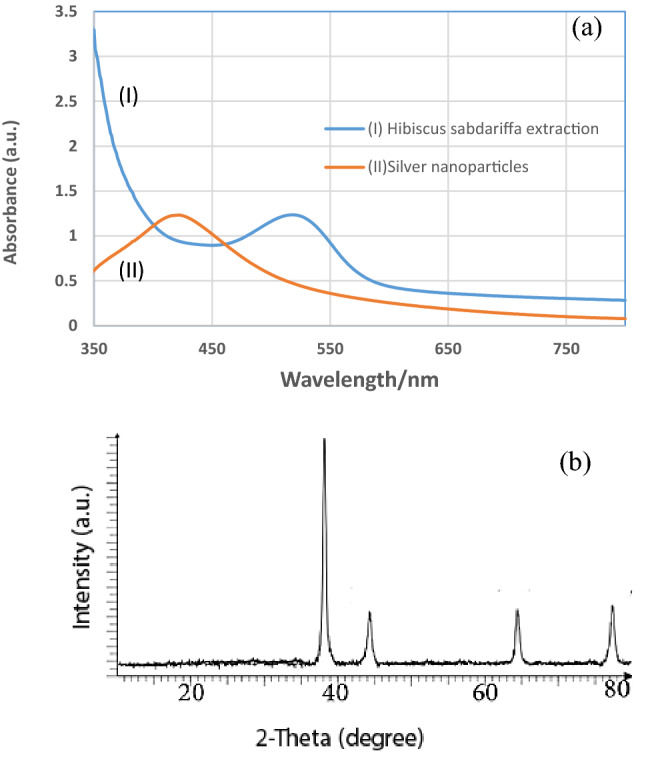
UV–Vis of the silver nanoparticles, UV–Vis of the *Hibiscus sabdariffa* extraction (**a**), XRD pattern of silver nanoparticles (**b**).

Ultraviolet–visible spectroscopy (UV–Vis) was used as a technique for gauging the formation of silver NPs in aqueous solutions. It functions through the excitation of surface plasmon resonance (SPR) of NPs. In fact, it is used for characterizing the formation of nanoparticles. Figure 2a shows the UV–Vis spectra of silver NPs and the appearance of a broad absorption peak from 400 to 500 nm^27^. The silver NPs formed after 40 min. Meanwhile, the broad peaks of the *Hibiscus sabdariffa* extraction were observed at 520 nm. The crystalline nature of silver NPs was confirmed by XRD analysis (Fig. 2b). The diffraction peaks were observed in the second range of 20–80. This information confirmed that the NPs had formed successfully. The XRD results clearly showed that the silver NPs which had formed were crystalline in nature and were in good agreement with the reported face-centered cubic (FCC) space group (JCPDS number 4-0787). Meanwhile, (111), (200), (220) and (311) crystallographic planes at 2θ of 38·3, 44·11, 64·0, and 77·34° were observed, respectively (Fig. 2b). The results of XRD confirmed aptly the reduction of silver ions to silver using the *Hibiscus sabdariffa* leaf extract. An estimate of the size of the particles was also made from the line broadening of the (111) reflection pattern using Scherer’s formula (D = Kl/b cos θ, where D is the average crystalline domain size perpendicular to the reflecting planes, l is the X-ray wavelength, b is the full width at half maximum and θ is the diffraction angle. The size of particles is an average of 8 nm.

**Figure 3:**
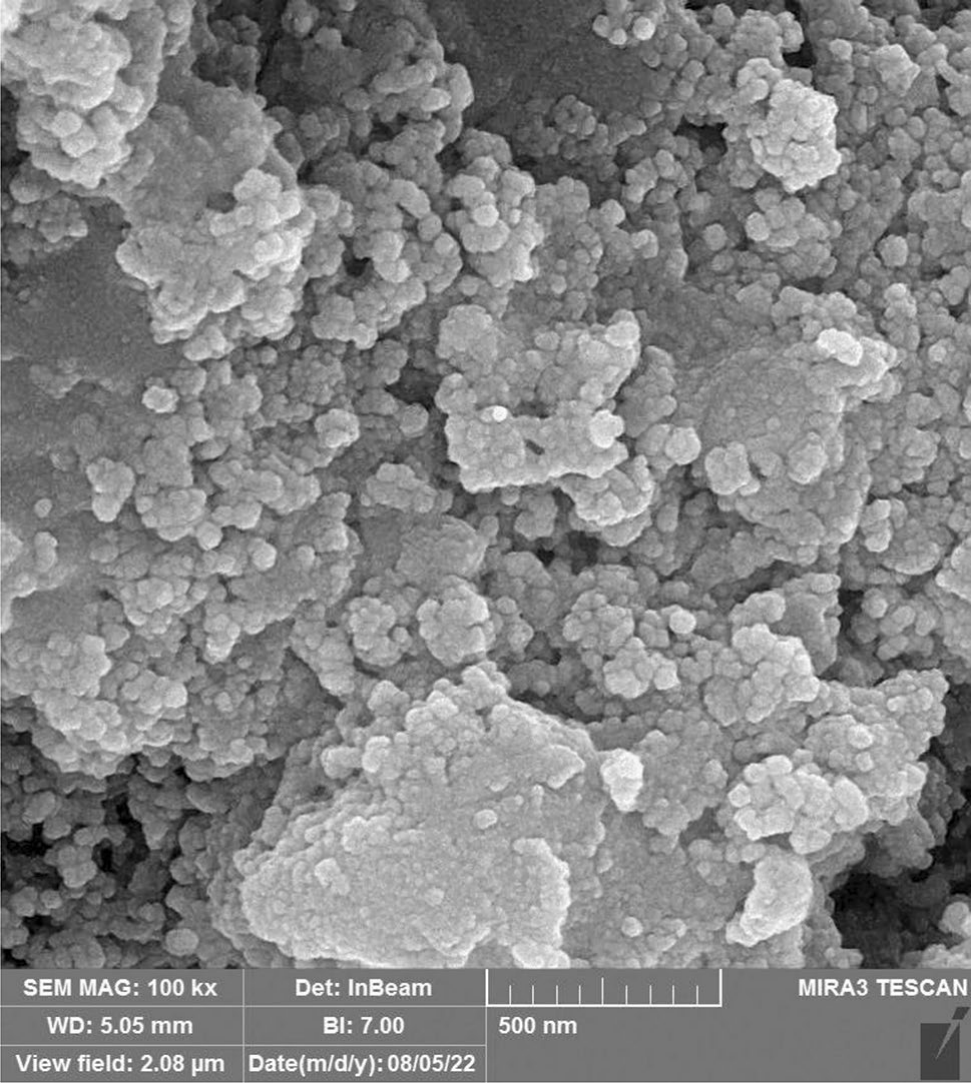
SEM image of silver nanoparticles.

**Figure 4:**
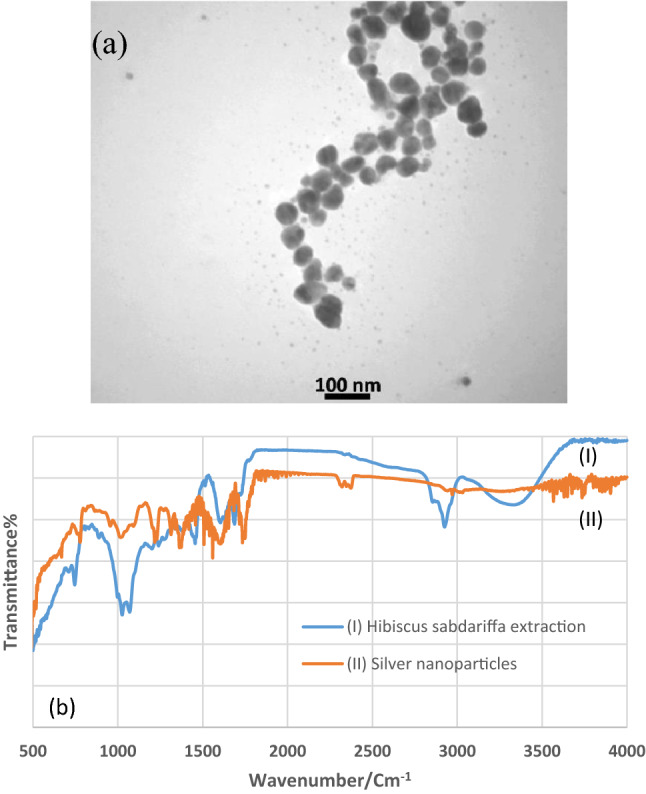
TEM image of synthesized silver nanoparticles using the *Hibiscus sabdariffa* extraction (**a**), FT-IR of silver nanoparticles, and the *Hibiscus sabdariffa* extraction (**b**).

The morphology and shape of bio-silver NPs were depicted using SEM (Fig. 3). Spherical shapes with sizes between 40 and 70 nm were confirmed. TEM image of silver NPs was considered for having the size and shape. Spherical shape with sizes between 10 and 50 nm were observed (Fig. 4a) (Zeiss—EM10C—100 kV). FT-IR of *Hibiscus sabdariffa* extraction and silver NPs formed using *Hibiscus sabdariffa* extraction (Fig. 4b). In the FT-IR spectrum of dried *Hibiscus sabdariffa*, the main peaks observed at ~ 1265/cm were attributed to the stretching of O–C acid groups^28^. Peaks between 1100 and 1071/cm indicated the presence of anthocyanins (cyanidin-3-O-sambubioside and delphinidin-3-O-sambubioside), showing the presence of these compounds^29^. The region between 3100 and 3500/cm is attributed to O–H stretch vibration^30^. Peaks at ~ 2919 and ~ 2848/cm refer to asymmetric and symmetrical stretching of CH_2_, respectively^31^. In the FT-IR of silver NPs spectrum, the strong and broad peak at 3434/cm represented a combined vibration frequency of –OH and –NH_2_ groups. The band at 1077/cm can be assigned to C–O stretching. The peak around 1387/cm can be assigned to C–N stretching of amine and amides groups, and the peak at 1637/cm is due to –C=C stretching^32^. At the FT-IR spectrum of silver NPs, the peaks relating to acid groups of plant and also anthocyanine were observed and others were related to silver NPs. According to FT-IR of silver NPs, the secondary structure of proteins and amino acids in the leaf extract as responsible factors for the reduction of Ag^+^ to form biomolecules encapsulated in stable silver NPs^33^.

### Seed germination and growth parameters

The green synthesis of silver NPs can improve plant tolerance to salinity. In general, the germination percentage decreased in response to higher concentrations of salinity. According to the results, by increasing the concentration of salinity, seed germination decreased (Table 1). The control treatment showed a germination percentage of 94%, although the percentage decreased to 25.31% by 180 mM salt (Table 1).

**Table 1:** The interactive effect of salinity stress and green nanoparticle on seed germination in Roselle.

Nano (mg/L)	Salinity levels (mM)								
	0	15	30	45	60	90	120	150	180
0	94 ± 5.78	86.6 ± 4.53	81.3 ± 1.32	74.6 ± 3.65	68.6 ± 2.89	64 ± 5.74	52.6 ± 4.55	43.3 ± 3.67	24.4 ± 3.92
15	94 ± 7.48	88 ± 3.21	79.3 ± 3.59	74 ± 2.67	66 ± 3.63	62.6 ± 5.29	52.7 ± 3.76	43.3 ± 2.98	22 ± 2.73
30	93.3 ± 3.47	89.5 ± 2.2	78.4 ± 2.74	72.6 ± 1.76	66 ± 1.38	64 ± 4.27	54 ± 4.77	43.3 ± 1.67	22.7 ± 2.37
45	90.7 ± 4.54	86.1 ± 4.4	81.7 ± 3.93	75.9 ± 4.65	74 ± 2.59	64.6 ± 4.29	58 ± 3.82	49.3 ± 4.42	34.3 ± 1.38
60	94.2 ± 6.43	86.6 ± 5.21	78 ± 4.88	72.2 ± 3.67	68.7 ± 4.52	62.4 ± 2.74	56 ± 2.79	41.3 ± 3.28	28 ± 2.67
75	94 ± 6.45	87.1 ± 1.32	79.5 ± 5.43	73.6 ± 1.77	65.3 ± 1.38	63.9 ± 1.11	54.2 ± 1.73	41.1 ± 5.38	22 ± 3.23
90	92.8 ± 2.13	85.3 ± 2.43	78 ± 1.89	74.6 ± 2.56	67.4 ± 3.76	62.2 ± 4.67	52.3 ± 5.28	38 ± 1.44	23.5 ± 2.61
LSD	2.1								

The results indicated that under 30, 45, 60, 120, 150, and 180 mM levels of salinity, 45 mg/L was the best concentration of silver NPs that could optimally mitigate the adverse effects of stress. However, according to the results, under 15 mM salinity, the highest percentage of germination was observed by 30 mg/L of silver NPs.

By increasing the concentration of salt from 0 to 120 mM, all seedling growth parameters decreased, including plant height, leaf and root fresh, and dry weights (Table 2). Under 60 and 120 Mm salt, the concentration of 90 mg/L of silver NPs significantly enhanced the plant height. The concentration of 90 mg/L of silver NPs was more effective in alleviating the adverse effects of 60 mM salinity on the leaf and root fresh and dry weights, compared with the other concentration (60 mg/L). However, under the effect of 120 mM salt, the effective concentration of silver NPs was largely dependent on plant characteristics. Both concentrations of 60 and 120 mM caused a decrease in RWC, although 90 mg/L silver NPs significantly improved the RWC.

**Table 2:** The interactive effects of sodium chloride (NaCl) salinity and silver nanoparticle on some growth characteristics of Roselle.

Treatments	0 mM NaCl	60 mM NaCl	120 mM NaCl
**Plant height (cm)**			
0	31.4 ± 3.3a	24.4 ± 5.4bc	19.7 ± 6.3c
45	32.4 ± 5.2a	26.8 ± 3.1ab	23.3 ± 4.4bc
90	31.4 ± 2.3a	30.1 ± 4.2a	27.7 ± 5.2ab
**Leaf fresh weight (g/plant)**			
0	1.16 ± 0.3ab	0.79 ± 0.23bc	0.65 ± 0.14c
45	1.17 ± 0.2ab	0.76 ± 0.12c	0.89 ± 0.25a–c
90	1.24 ± 0.2a	0.82 ± 0.21bc	0.87 ± 0.18a–c
**Leaf dry weight (g/plant)**			
0	0.26 ± 0.05a	0.22 ± 0.06a	0.16 ± 0.03a
45	0.30 ± 0.09a	0.19 ± 0.04a	0.19 ± 0.03a
90	0.28 ± 0.07a	0.21 ± 0.02a	0.20 ± 0.02a
**Root fresh weight (g/plant)**			
0	1.67 ± 0.12a	1.27 ± 0.25c	0.75 ± 0.22e
45	1.79 ± 0.42a	1.32 ± 0.18bc	0.86 ± 0.27ed
90	1.78 ± 0.24a	1.45 ± 0.32b	1.00 ± 0.31d
**Root dry weight (g/plant)**			
0	0.19 ± 0.04b	0.14 ± 0.03b	0.08 ± 0.01b
45	0.18 ± 0.03b	0.16 ± 0.04b	0.65 ± 0.14a
90	0.19 ± 0.01b	0.19 ± 0.02ab	0.11 ± 0.02b
**Relative water content (%)**			
0	82.7 ± 6.9a	70.6 ± 6.6d	61.3 ± 6.1e
45	83 ± 8.1a	72.9 ± 3.5d	71.5 ± 7.5d
90	82.1 ± 5.8ab	77.7 ± 4.1bc	74.6 ± 4.6cd

Within a column, means followed by the same letter are not significantly different (P < 5).

### Antioxidant enzyme activity, proline, and soluble sugar

**Figure 5:**
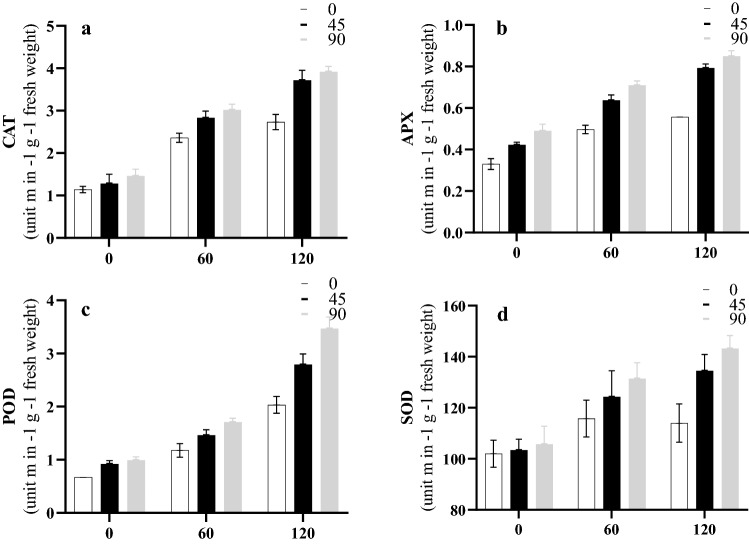
Interactions effects of salinity and NPs on CAT (**a**), APX (**b**), POD (**c**), and SOD (**d**).

According to the results of ANOVA, the interaction effect of salinity and NPs was statistically significant on all antioxidant enzymes, including CAT, APX, POD, and SOD. The highest mean values of CAT, APX, POD, and SOD were observed in plants treated with 120 mM NaCl (Fig. 5). In addition, by increasing the concentration of NPs, the values of enzymes increased under the effects of the three salt stress levels. According to the current study, both concentrations of NPs had significant effects on CAT, APX, POD, and SOD activities in plants under salt stress.

The lowest mean values of proline and soluble sugar contents (0.26 and 4.4 mg/g dry matter, respectively) were observed in the control treatment (Table 3). However, the highest mean values were observed in plants exposed to 120 mM NaCl and 90 mg/L nanoparticles. Similar to antioxidant enzymes, by increasing both concentrations of salt and nanoparticles, the proline and soluble sugar contents increased. Under salinity stress, NPs caused an increase in the accumulation of proline and soluble sugars in the leaves. According to the results, NPs alleviated the adverse effects of salinity by antioxidant enzymes, proline, and soluble sugar contents.

**Table 3:** Effect of salinity stress and nanoparticle interaction on shoot proline and soluble sugar content in Roselle.

Nanoparticle (mg/L)	Proline (mg/g dry matter)			Soluble sugar content (mg/g dry matter)		
	NaCl (mM)			NaCl (mM)		
	0	60	120	0	60	120
0	0.26 ± 0.1e	0.45 ± 0.15de	0.77 ± 0.12bc	4.4 ± 0.26e	9.5 ± 2.67d	15.1 ± 4.14b
45	0.30 ± 0.09e	0.56 ± 0.17 cd	1 ± 0.29b	4.6 ± 0.33e	10.6 ± 3.12d	16 ± 4.78b
90	0.28 ± 0.11e	0.62 ± 0.16 cd	1.26 ± 0.47a	4.6 ± 0.38e	12.4 ± 3.23c	18.7 ± 3.72a

### Flavonoid and anthocyanin contents

**Figure 6:**
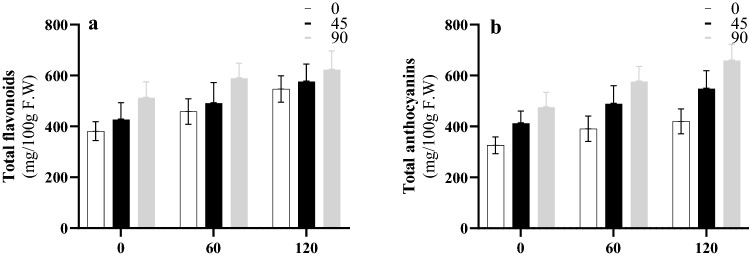
Interactions effects of salinity and NPs on total flavonoids (**a**) and anthocyanin content (**b**) in Roselle.

In the present study, flavonoids and anthocyanin contents were also measured. The results showed that the interaction of silver NPs and salt stress had significant effects on the flavonoids and the anthocyanin contents. Increasing the concentration of silver NPs from 0 to 90 mg/L was associated with an increase in flavonoids and anthocyanin contents (Fig. 6). In a similar way, the results revealed that an increase in salt concentration from 0 to 120 mM significantly increased the amounts of flavonoids and anthocyanin contents from 381 to 547 mg/100 g F.W, and from 326 to 420 mg/100 g F.W, respectively. Among all treatments, the highest flavonoids and anthocyanin contents (623 and 659 mg/100 g F.W, respectively) occurred in response to the interaction between 90 mg/L silver NPs and 120 mM salt stress.

**Figure 7:**
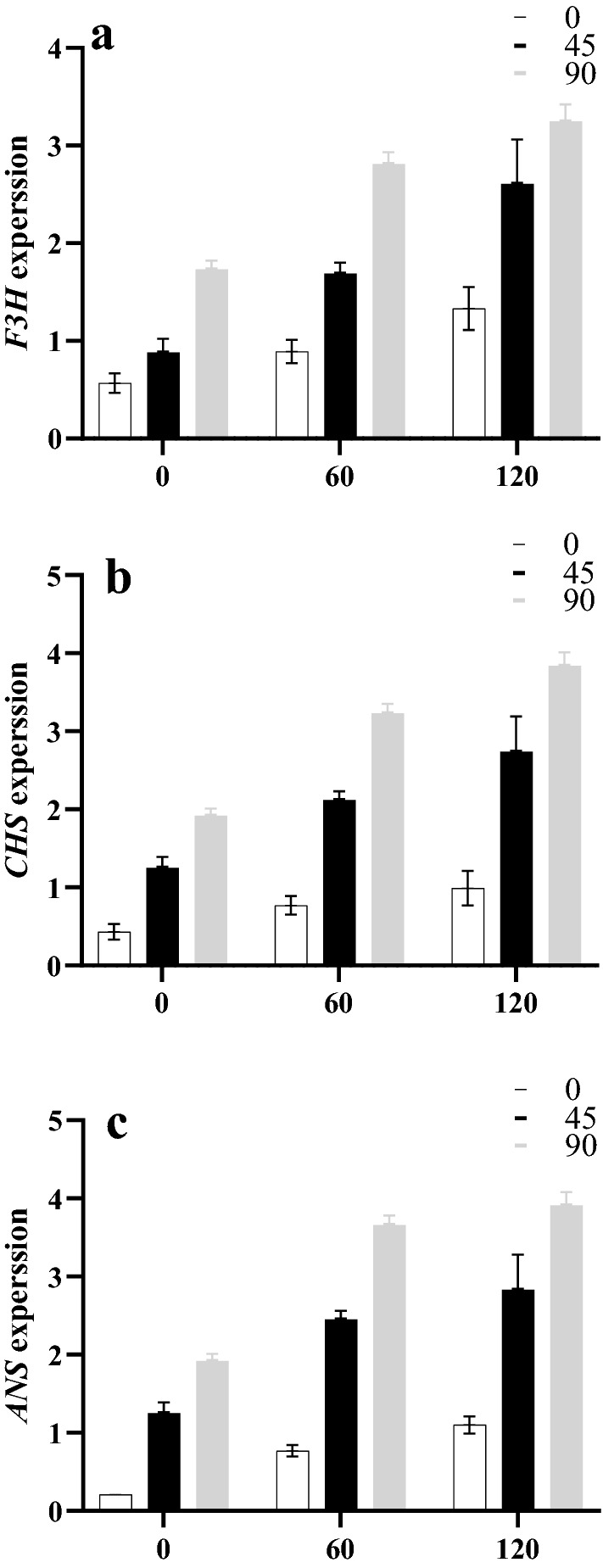
Interactions effects of salinity and NPs on expression of *F3H*, *CHS*, and *ANS* genes in Roselle.

**Figure 8:**
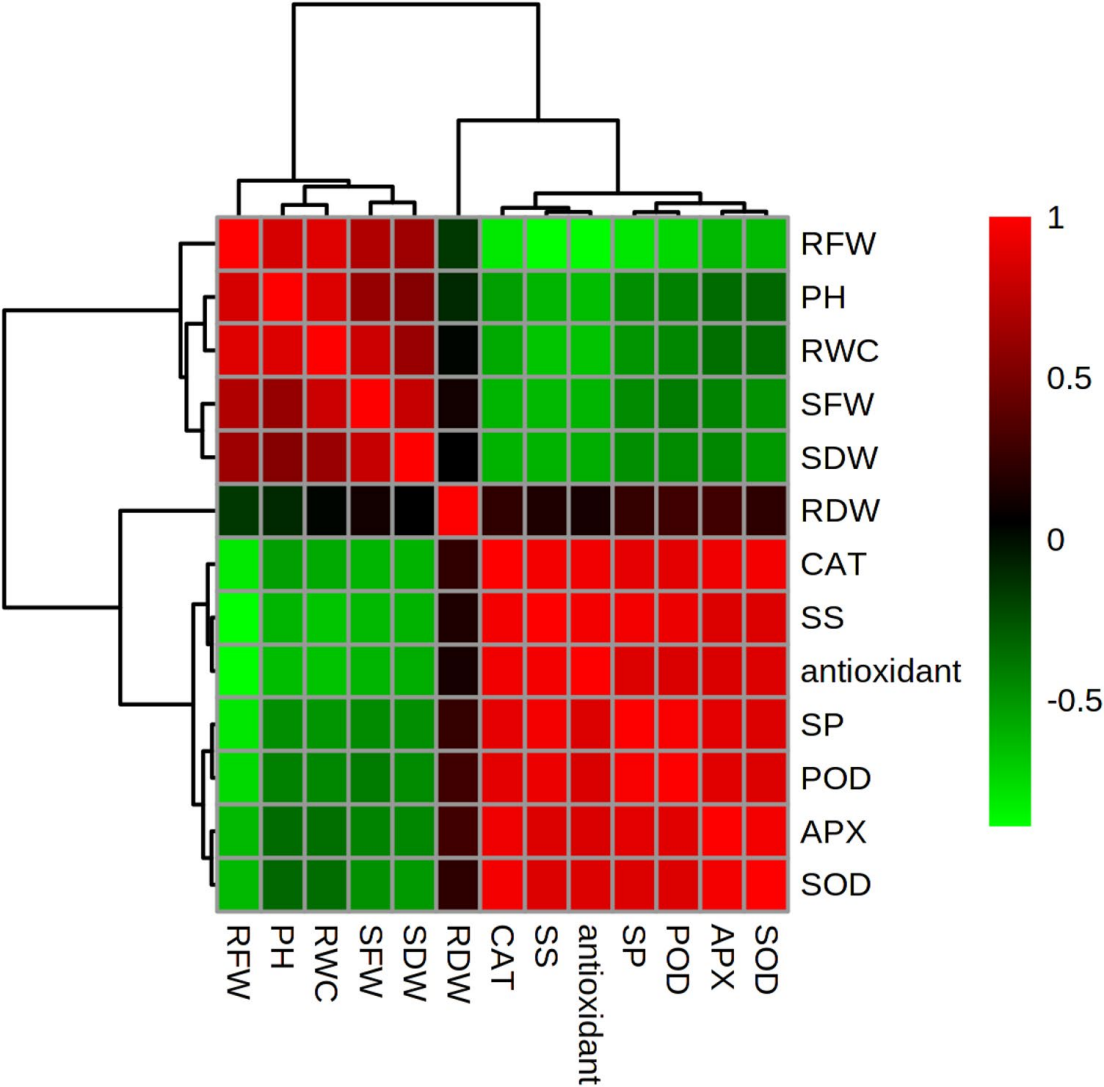
Heat map correlation among the measured characters of Roselle (*PH* plant height, *SFW* shoot fresh weight, *SDW* shoot dry weight, *RFW* root fresh weight, *RDW* root dry weight, *SP* shoot proline, *SS* shoot soluble sugar content). The color scales represent the values were correlation coefficients from − 1 (green) to 1 (red).

The *F3H*, *CHS*, and *ANS* genes are involved in the biosynthesis of flavonoids and anthocyanin. In the present research, the expressions of *F3H*, *CHS*, and *ANS* genes were studied. Our results demonstrated that the expected amplicon size for the *F3H, CHS*, and *ANS* genes were 84, 141, and 89 bp, respectively (Supplementary Table S1). The results showed that the interaction of the silver NPs and salt stress had significant effects on the three gene expressions. According to the results, the expression of *F3H*, *CHS*, and *ANS* genes had similar patterns (Fig. 7). The expression of the genes significantly increased by a rise in the concentrations of salt and silver NPs. In this regard, the applications of 90 mg/L silver NPs caused significantly higher levels of expression by the genes compared to other treatments. The lowest levels of gene expression (about 0.43, 0.57, and 0.21 times the normal level, regarding the *CHS* and *F3H* and *ANS* genes, respectively) were obtained in the control group. According to the results of heat map correlation analysis, significant correlations were observed between several traits in Roselle (Fig. 8). Furthermore, the results indicated that a significant correlation existed among enzyme activity, RWC, shoot and root fresh weights. In addition, a significant correlation was observed between the RWC and plant height.

## Discussion

Due to water shortage and climate change, salt stress is increasingly becoming a threat to agricultural products^34^. The present study was designed to determine the interaction effects of NaCl and synthetic silver NPs on seed germination, seedling growth parameters, antioxidant enzymes, and proline, soluble sugar, flavonoid, and anthocyanin contents of Roselle.

Silver NPs help plants regulate physiological functions such as nitrogen metabolism, photosynthesis, the expression of genes, the production of aquaporins, and the mechanism of water uptake during seed germination^35^. The results of the current study showed that NPs enhanced seed germination under salinity stress. Previous studies demonstrated the noticeable roles of NPs in regulating seed germination in many plants under different abiotic stress^36^. Baz et al.^37^ reported that soluble NPs significantly enhanced the seed germination of lettuce under salinity stress. DeRosa et al.^38^ reported that silica NPs (silicon dioxide, SiO_2_) can assist plants in developing a protective layer on their cell walls and, thus, overcome the salinity-induced stress to a certain extent. In addition, NPs penetrate the seed coat and facilitate water uptake by seeds, thereby resulting in a dramatic increase in seed germination^35^. In the second experiment of the present study, the interaction effects between salinity stress and NPs on the growth parameters of Roselle were evaluated. According to the results, the NPs improved plant growth indices, while increasing their resistance to salinity stress. Gopinath et al.^39^ reported the use of AuNPs in *Gloriosa superb* which assisted in increasing the plant height, the number of leaves, leaf area, chlorophyll, and sugar contents, ultimately increasing plant yield*.* These effects of NPs could be due to their physicochemical properties which could potentially improve plant metabolisms^40^. In addition, NPs are able to enter plant cells and leaves, thereby facilitating plant growth and development^41^. Plant growth indices can be improved by nano-SiO_2_, AgNPs, sulfur NPs, TiO_2_ NPs, iron oxide NPs, CuO NPs, wsCNTs, and ZnO NPs^41^.

Based on the results, the interaction effects of NPs and salinity significantly increased the amounts of proline and soluble sugars. These are important as natural solutes that maintain cellular homeostasis and assist in cell osmoregulation under salinity stress. Similar to our findings, Khalofah et al.^42^ reported that NPs increased the amounts of proline and soluble sugars in linseed under salt stress. Abiotic types of stress such as salinity and drought caused excessive amounts of reactive oxygen species (ROS), which resulted in the vulnerability of proteins, DNA, and carbohydrates in plants. One of the most important mechanisms of plants that can maintain their proper and sustainable development is a line of defense mechanism by enzymatic antioxidants. Some of these principal enzymatic antioxidants are CAT, SOD, POD, and APX. The activities of CAT, APX, POD, and SOD vary expansively, depending on whether or not the seedlings are exposed to NPs when salinity stress exists. These enzymes are vital for the defense system against ROS in plants. Here, enzymatic activities generally increased in response to silver NPs. A previous study revealed that TiO_2_NPs can increase the activity of antioxidant enzymes, including CAT, POD, and SOD, thereby protecting chloroplasts in spinach from excessive sunlight^43^. Furthermore, ample amounts of evidence confirm the positive role of NPs in increasing the levels of antioxidant activity. Kumar et al.^44^ reported that Au-NPs strengthen the antioxidant system in *Arabidopsis thaliana* while improving the expression of micro-RNAs. Increasing the activities of CAT, SOD, POD, and APX enzymes showed that the line of a defense mechanism by antioxidants was one of the most important mechanisms of Roselle that contributed to the maintenance of its proper, sustainable development, which might explain enhancements in growth parameters^45^.

Salt stress can reduce the amounts of flavonoids and anthocyanin in susceptible plant species^46^. In contrast, there are other species that respond differently to salt stress, and the amounts of flavonoids and anthocyanin compounds usually increase in those species^47^. In the present study, by increasing the concentrations of both salt and green silver NPs, the flavonoids and anthocyanin compounds accumulated in the leaves of Roselle. Furthermore, the results of real-time PCR showed that the expression of the *CHS*, *F3H* and *ANS* genes increased in response to salt stress and green silver NPs. Dihydrokaempferol is one of the most fundamental substances which is active in the pathways that synthesize flavonoids and anthocyanin. It is produced by the activity of CHS and F3H enzymes^15^. It seems that the up-regulation of *CHS* and *F3H* genes can directly increase the synthesis of dihydrokaempferol. In the same line, the application of green silver NPs led to an up-regulation of the *ANS* gene as an important factor involved in the biosynthetic pathway of anthocyanin, which transforms colorless leucoanthocyanidins into colored anthocyanidins. Finally, this increased the production of total flavonoids and anthocyanin contents. These results were in accordance with similar ones reported previously in the literature^48^, where silver or zinc NPs caused an increase in anthocyanin content.

There were relationships among the amounts of flavonoids, anthocyanin compounds and the antioxidant capacity of Roselle leaf samples of the seedlings. The total peroxidase activity showed significant oxidation of phenolic compounds, such as flavonoids and anthocyanins. Furthermore, the coexistence of POD and phenolic compounds can act as an H_2_O_2_-scavenging system^49^. In the current study, phenolic compounds accumulated and the POD activity increased in the leaves of Roselle seedlings in response to salt stress. Higher amounts of H_2_O_2_ in plant cells can be parallel to the accumulation of oxidation among phenolic compounds^49^. Accordingly, it seems that the rise in flavonoids and anthocyanin can be another mechanism of salt tolerance in Roselle plants, while NPs contributed to this mechanism and the antioxidant defense mechanism.

## Conclusion

Salinity adversely affected growth parameters and seed germination in Roselle plants. The results demonstrated that the applied green silver NPs generally alleviated the adverse effects of salinity stress by empowering the defense mechanisms, especially antioxidant enzymes and accumulations of soluble sugars and proline. It can be concluded that the application of NPs significantly ameliorated the harmful effects of salinity stress.

## Supplementary Information

Supplementary Table S1.

## Data Availability

All data analyzed during this study are included in this published article and the supplementary information files.
