# Supplementary material for: Alleviating the adverse effects of salinity on Roselle plants by green synthesized nanoparticles
Source: Sci Rep. 2022 Oct 28;12:18165. doi: 10.1038/s41598-022-22903-9 (PMC9616842; doi:10.1038/s41598-022-22903-9)
Supplement: Supplementary file 1 — Supplementary Table S1. [file 41598_2022_22903_MOESM1_ESM.docx]

**Supplementary data**

**Alleviating the adverse effects of salinity on Roselle plants by green synthesized nanoparticles**

Mohammad Sadat-Hosseini ^a*^, Atena Naeimi ^b^, Naser Boroomand ^c^, Mostafa Aalifar ^d^ and Mostafa Farajpour ^e*^

^a^ Department of Horticulture Science, Faculty of Agriculture, University of Jiroft, Jiroft, Iran.

^b^ Department of Chemistry, Faculty of Science, University of Jiroft, Jiroft, Iran.

^c^ Department of Soil Science, Faculty of Agriculture, Shahid Bahonar University of Kerman, Kerman, Iran.

^d^ Young Researchers and Elite Club, Hamedan Branch, Islamic Azad University, Hamedan, Iran.

^e^ Crop and Horticultural Science Research Department, Mazandaran Agricultural and Natural Resources Research and Education Center, Agricultural Research, Education, and Extension Organization (AREEO), Sari, Iran.

***Corresponding authors E-mails:**

[Farajpour_m@ut.ac.ir](mailto:Farajpour_m@ut.ac.ir) (Mostafa Farajpour)

[m.hosseini@ujiroft.ac.ir](mailto:m.hosseini@ujiroft.ac.ir) (Mohammad Sadat-Hosseini)

**Table S1**

Primer sequence

| Gene | Primer pairs | Primer sequences (5'-3') | Expected amplicon size (bp) | AT (C) | Gene accis |
| --- | --- | --- | --- | --- | --- |
| *F3H* | F  R | TTACCACCGGACATGTCGAA  CATTTCCGACATGACCCGTC | 84 | 58 | KR709157 |
| *CHS*  *ANS* | F  R  F  R | CCTTCTTCCCTTGATTTCTTCCTC  GGACCAAGTAGAATCCAAGTTAGC  AGTTGAAGAAAGCCGCCATG  AGCTTTCCTGACACGATCCA | 141  89 | 59  59 | KR709156  KX818221 |
| *18S rRNA* | F  R | CGACCACCTTTTCATTCAGATTCC  GATCATTGTCGAAACCTGCCTAG | 191 | 59 | KP262033 |
